# Effectiveness of transcranial alternating current stimulation for controlling chronic pain: a systematic review

**DOI:** 10.3389/fneur.2023.1323520

**Published:** 2023-12-20

**Authors:** Min Cheol Chang, Marie-Michèle Briand, Mathieu Boudier-Revéret, Seoyon Yang

**Affiliations:** ^1^Department of Rehabilitation Medicine, College of Medicine, Yeungnam University, Daegu, Republic of Korea; ^2^Division of Trauma, Research Center, Hôpital du Sacré-Cœur de Montréal, CIUSSS du Nord-de-l’Île-de-Montréal, Montréal, QC, Canada; ^3^Department of Physical Medicine and Rehabilitation, Hôpital du Sacré-Cœur de Montréal, CIUSSS du Nord-de-l’Île-de-Montréal, Montréal, QC, Canada; ^4^Faculty of Medicine, Université de Montréal, Montréal, QC, Canada; ^5^Department of Physical Medicine and Rehabilitation, University of Montreal Health Center, Montréal, QC, Canada; ^6^Department of Rehabilitation Medicine, School of Medicine, Ewha Woman's University Seoul Hospital, Seoul, Republic of Korea

**Keywords:** transcranial alternating current stimulation, chronic pain, fibromyalgia, low back pain, migraine, treatment, review

## Abstract

**Background:**

Chronic pain is common, disruptive, and often treatment-resistant. Hence, researchers and clinicians seek alternative therapies for chronic pain. Transcranial alternating current stimulation (tACS) is an emerging neuromodulation technique that non-invasively modulates neural oscillations in the human brain. tACS induces pain relief by allowing the neural network to restore adequate synchronization. We reviewed studies on the effectiveness of tACS in controlling chronic pain.

**Methods:**

The PubMed, SCOPUS, Embase, and Cochrane Library databases were systematically searched for relevant studies published until December 6, 2023. The key search phrase for identifying potentially relevant articles was [(Transcranial Alternating Current Stimulation OR tACS) AND pain]. The following inclusion criteria were applied for article selection: (1) studies involving patients with chronic pain; (2) tACS was applied for controlling pain; and (3) follow-up evaluations were performed to assess the degree of pain reduction after the application of tACS.

**Results:**

We identified 2,330 potentially relevant articles. After reading the titles and abstracts and assessing eligibility based on the full-text articles, we included four articles in our review. Among the included studies, tACS was used for fibromyalgia in one study, low back pain (LBP) in two studies, and migraine in one study. In the study on fibromyalgia, it did not show a better pain-reducing effect of tACS compared with sham stimulation. Two studies on LBP showed conflicting results. In migraine, tACS showed a positive pain-reducing effect 24–48 h after its application.

**Conclusion:**

There is insufficient research to draw a conclusive judgment on the effectiveness of tACS in controlling chronic pain. More studies across various chronic pain-related diseases are required for a definitive conclusion.

## Introduction

Chronic pain is pain that persists for more than 3 months or after complete healing, which is a leading cause of disability and disease worldwide (1, 2). Approximately 20% of the adult population experiences chronic pain, with 8% of individuals reporting severe pain that disrupts their life and work activities (3). To manage chronic pain, various therapeutic methods have been applied, including physical therapy, psychotherapy, medication, procedures, and surgery (1, 2). However, despite these treatments, chronic pain often remains uncontrolled.

The brain plays a fundamental role in the processing of pain (4). Several previous studies using electroencephalography (EEG) and magnetoencephalography (MEG) demonstrated that chronic pain is closely associated with abnormal neuronal oscillations (5–8). The peak frequency of neuronal oscillations measured by EEG or MEG was lower in patients with chronic pain compared with that of healthy controls (5–8). Specifically, changes in neural oscillations at gamma (30–100 Hz) frequencies in prefrontal brain area are related to chronic pain (9). The modulation of neural oscillations has been suggested to be a promising novel therapeutic approach for controlling chronic pain.

Transcranial alternating current stimulation (tACS) is an emerging neuromodulation technique that non-invasively modulates neural oscillations in the human brain (10–12). During the application of tACS, a weak alternating sinusoidal current is administered to the scalp with the aim of synchronizing neural oscillations at the stimulation frequency, thereby enhancing their amplitude and causing a new balance by forcing the neural network to restore adequate synchronization (10–12). This neural modulation by tACS was proposed to induce pain relief.

Thus far, some clinical trials have been conducted to investigate whether tACS has a pain-reducing effect in patients with chronic pain (13–17). In this study, we review previous studies on the effectiveness of tACS in controlling chronic pain and integrate their results to draw a comprehensive conclusion on the therapeutic possibility of tACS for pain reduction.

## Methods

This systematic review conformed to the recommendations of the Preferred Reporting Items for Systematic Review and Meta-analysis. The protocol was registered on the international platform of registered systematic reviews protocols (registration number: INPLASY2023120012).

The population, intervention, comparison, and outcome (PICO) setting of the current systematic review was as follows: P: patients with chronic pain; I: tACS combined with or without other therapies for controlling pain; C: placebo or sham stimulation; and O: pain intensity. Two authors (MC and SY) searched for relevant studies published until December 6, 2023, in the PubMed, SCOPUS, Embase, and Cochrane Library databases (Supplementary material 1). The key search phrase for identifying potentially relevant articles was [(Transcranial Alternating Current Stimulation OR tACS) AND pain]. The following inclusion criteria were applied for the selection of articles: (1) patients with chronic pain; (2) tACS was applied for controlling pain; and (3) follow-up evaluations were performed after tACS stimulation to assess the degree of pain reduction after the application of tACS. We excluded the following studies: (1) review articles; (2) animal studies; and (3) conference abstracts or presentations.

After duplicate publications were deleted, two reviewers (MC and SY) independently evaluated potentially eligible studies that were identified by our search. Articles were screened for eligibility based on a review of the title and abstract, and disagreements were resolved by consensus. The full texts of eligible articles were accessed and read independently by the two reviewers (MC and SY).

The risk of bias of selected studies was evaluated using the criteria described in the Cochrane Handbook for Systematic Reviews of Interventions to assess potential bias (18). The domains to evaluate the risk of bias were as follows: (1) random sequence generation and allocation concealment (selection bias); (2) blinding of participants and personnel (performance bias); (3) blinding of outcome assessment (detection bias); (4) incomplete outcome data (attrition bias); (5) selective reporting (reporting bias); and (6) other biases. Two independent reviewers performed these evaluations (MC and SY), and discrepancies were resolved through discussion.

## Results

### Search results

**Figure 1:**
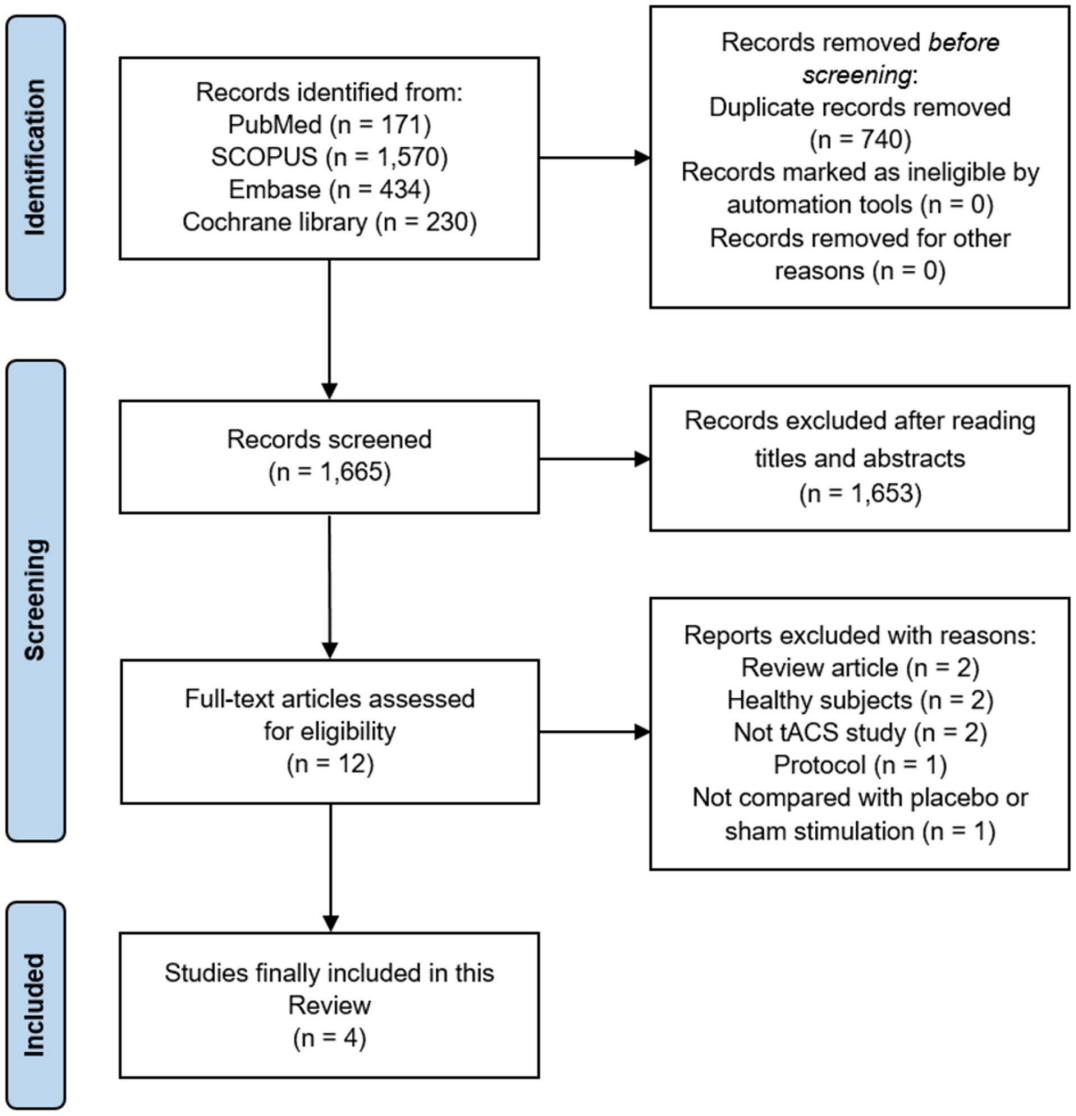
PRISMA flow diagram. tACS, Transcranial alternating current stimulation.

A total of 2,405 articles were identified using the search terms. Of these, 740 duplicates were excluded from further analysis. After reading the titles and abstracts, 1,653 articles were excluded because they did not meet the inclusion criteria. Subsequently, 12 full-text articles were retrieved to verify study eligibility, and a total of four publications were finally included in this review (Figure 1) (13, 14, 16, 17). These four publications were all randomized controlled trials (RCTs) (13, 14, 16, 17). Among these four studies, two were conducted under a randomized, double-blind, crossover design (13, 17). Table 1 presents the characteristics of the included articles, and Table 2 presents the application methods for tACS and combined treatments with tACS. Among four included studies, tACS was applied for fibromyalgia in one study (16), low back pain (LBP) in two studies (13, 17), and migraine in one study (14).

**Table 1:** Characteristics of the included studies.

#	First author	Published year	No. of patient	Age, years (mean ± SD)	Disease	Pain duration of patients	Study type	Outcome parameters	Results
1	Lin (16)	2022	35 (tACS: sham = 18:17)	tACS: 48.3 ± 13.6	Fibromyalgia	≥3 months	RCT	NRS, FIQ, BAI, BDI-II, and PSQI	All the measured outcomes were not significantly different between the tACS and sham stimulation groups.
				Sham: 48.9 ± 12.3					
2	Ahn (13)	2019	20	Age range: 18–65, no information on mean	Low back pain	≥5 months	Randomized, double-blind, crossover design	DVPRS, ODI, and EEG	The DVPRS score was significantly lower after tACS than after sham stimulation, but the ODI was not. The increase in alpha oscillations was significantly higher after tACS compared to sham stimulation.
3	Prim (17)	2019	20	43.0 ± 13.4	Low back pain	≥6 months	Randomized, double-blind, crossover design	DVPRS	No significant difference was found between tACS and sham stimulation.
4	Antal (14)	2020	25 (tACS: sham = 16:9)	tACS: 31.1 ± 8.9	Migraine	≥6 months	RCT	NRS	The reduction of NRS at 2 and 4 h after stimulation was significantly higher in the tACS group than in the sham stimulation group.
				Sham: 28.1 ± 10.5					

tACS, Transcranial alternating current stimulation; SD, Standard deviation; RCT, Randomized controlled trial; NRS, Numeric rating scale; FIQ, Fibromyalgia impact questionnaire; BAI, Beck Anxiety Inventory; PSQI, Pittsburgh Sleep Quality Index; DVPRS, Defense and Veterans Pain Rating Scale; ODI, Oswestry Disability Index; and EEG, electroencephalogram.

**Table 2:** Application methods for tACS treatment and combined treatments with tACS.

#	First author	Simulation site	Intensity (mA)	Duration (min)	Frequency (Hz)	No. of sessions	Combined treatments with tACS	Treatment received by control group
1	Lin (16)	M1	1	20	50	10	No combined treatment.	Sham stimulation
2	Ahn (13)	Bilateral F-lobe (F3, F4)	1	40	10	1 per study arm	No combined treatment.	Sham stimulation
3	Prim (17)	M1	1	40	10	1 per study arm	No combined treatment.	Sham stimulation
4	Antal (14)	O-lobe (Oz)	0.4	15	140	1 (at the beginning of the migraine attack)	The included patients were allowed to take their regular acute migraine medications	Sham stimulation and regular acute migraine medications

tACS, Transcranial alternating current stimulation; M1, Primary motor cortex; F-lobe, Frontal lobe; and O-lobe, Occipital lobe.

### Fibromyalgia

In 2022, Lin et al. conducted an RCT to evaluate the effect of tACS on the left primary motor cortex (M1) for the management of chronic pain from fibromyalgia (16). Thirty-eight patients having a history a widespread pain with ≥3 months duration were recruited and randomly allocated to the tACS (*n* = 19) and sham stimulation (*n* = 19) groups. tACS was conducted with a daily session of 20 min of stimulation of 1 mA at 50 Hz (duty cycle with an on time of 2 s and off time of 8 s) over the left M1 (C3 position in the 10/20 system for the EEG electrode positions) for 10 sessions in 2 weeks. For the administration of the electrical current, a 4 × 1 ring electrode configuration was used. The pain severity and physical function were assessed using the numeric rating scale (NRS) and the fibromyalgia impact questionnaire (FIQ) at baseline and after 2 weeks of tACS treatment. Additionally, the Beck Anxiety Inventory, Beck Depression Inventory-II, and Pittsburgh Sleep Quality Index (PSQI) were checked. During the study, one patient in the tACS group and two in the sham stimulation group dropped out. After 10 sessions of tACS and sham stimulation, there were no significant differences in the changes in all the measurements between the groups. After tACS, various adverse events, including headache, scalp pain, stinging, itch, burning sensation, drowsiness, and difficulty concentrating, were reported. However, the severity of all the adverse events was mild, and the occurrence rate was not different from the sham stimulation group.

### Low back pain

In 2019, Ahn et al. (13) performed a randomized, double-blind, crossover design study to investigate the effect of tACS on controlling LBP. They recruited 20 patients with chronic LBP persisted at least 5 months and randomly allocated them into two groups (tACS and sham stimulation groups). After the first session was completed, all the patients had a washout interval of 1–3 weeks. They were then crossed over to the other groups and had a second session. Two stimulating electrodes (5 cm × 5 cm each) were placed at the bilateral frontal lobe (F3 and F4) and delivered a sinusoidal waveform with 1 mA amplitude for 40 min. The return electrode (5 cm × 7 cm) was placed at Pz (medial parietal region). At pretreatment and after completing each session, the Defense and Veterans Pain Rating Scale (DVPRS, from 0 to 10, 0 = no pain and 10 = worst imaginable pain, similar to the NRS), Oswestry Disability Index (ODI), and enhancement of alpha oscillation were measured. After tACS, the pain severity measured by DVPRS was significantly reduced compared to sham stimulation, but ODI was not. The increase in alpha oscillations was significantly higher after tACS compared to sham stimulation.

In the same year, Prim et al. (17) conducted a randomized, double-blind, crossover design study in 20 patients with chronic LBP (pain duration: at least 6 months). The included patients were divided into two groups (10 patients per group). The schedule for the application of tACS, areas that place the electrodes, and electrode type were the same as in Ahn et al.’s study on the forehead. Prim et al. defined the responders as patients who had a decrease of ≥2 points on the DVPRS after completing the stimulation session. Twice as many patients reported being responders after tACS treatment vs. after sham stimulation, but no significant difference was found. They also checked the degree of adverse effects, including headache, neck pain, scalp pain, tingling, itching, ringing/buzzing noise, burning sensation, local redness, sleepiness, trouble concentrating, improved mood, worsening of mood, dizziness, and flickering lights, with numeric scores. The significant difference in score for each side effect was not shown between tACS and sham stimulation.

### Migraine

In 2020, Antal et al. (14) recruited 40 patients with chronic migraine (pain duration: ≥6 months), and they were randomly allocated to the tACS (25 patients) and sham stimulation (15 patients) groups. Among these patients, 25 patients—16 in the tACS group and nine in the sham stimulation group—completed the study. The study was conducted over the course of 6 weeks. Patients were asked to write a headache diary during the study period. During the study, the frequency of the migraine attacks, duration of the pain, and use of analgesics were recorded. The pain degree was also measured with NRS at the onset of a migraine attack as well as 1, 2, 4, 8, 24, and 48 h thereafter. tACS or sham stimulation was applied by the patient at home. The stimulation was applied at the beginning of the migraine attack. The stimulating electrode (4 cm × 4 cm) was placed over the occipital lobe (Oz) and the return electrode (5 cm × 7 cm) over the Cz electrode position. tACS with 0.4 mA was applied for 15 min. During the study, 65 migraine attacks were treated in 16 patients in the tACS group (mean: 4.06 attacks/patient) and 37 in nine patients in the sham stimulation group (mean: 4.11 attacks/patient). In the tACS group, 27 of the 65 migraine attacks were treated with oral medications within 2 h after the stimulation, compared to 14 of the 37 attacks in the sham stimulation group. During the migraine attacks without taking oral medications, the pain disappeared within 2 h post-stimulation in 14 of the 38 attacks in the tACS group but in none of the 23 attacks in the sham stimulation group, with a statistically significant intragroup difference. The rate of terminated migraine attacks that did not require acute rescue medication was significantly higher in the tACS group (21.5%) than in the sham stimulation group (0%). The pain severity measured with NRS was significantly lower after tACS than after sham stimulation in 2 and 4 h after the stimulation.

### Risk of Bias

**Figure 2:**
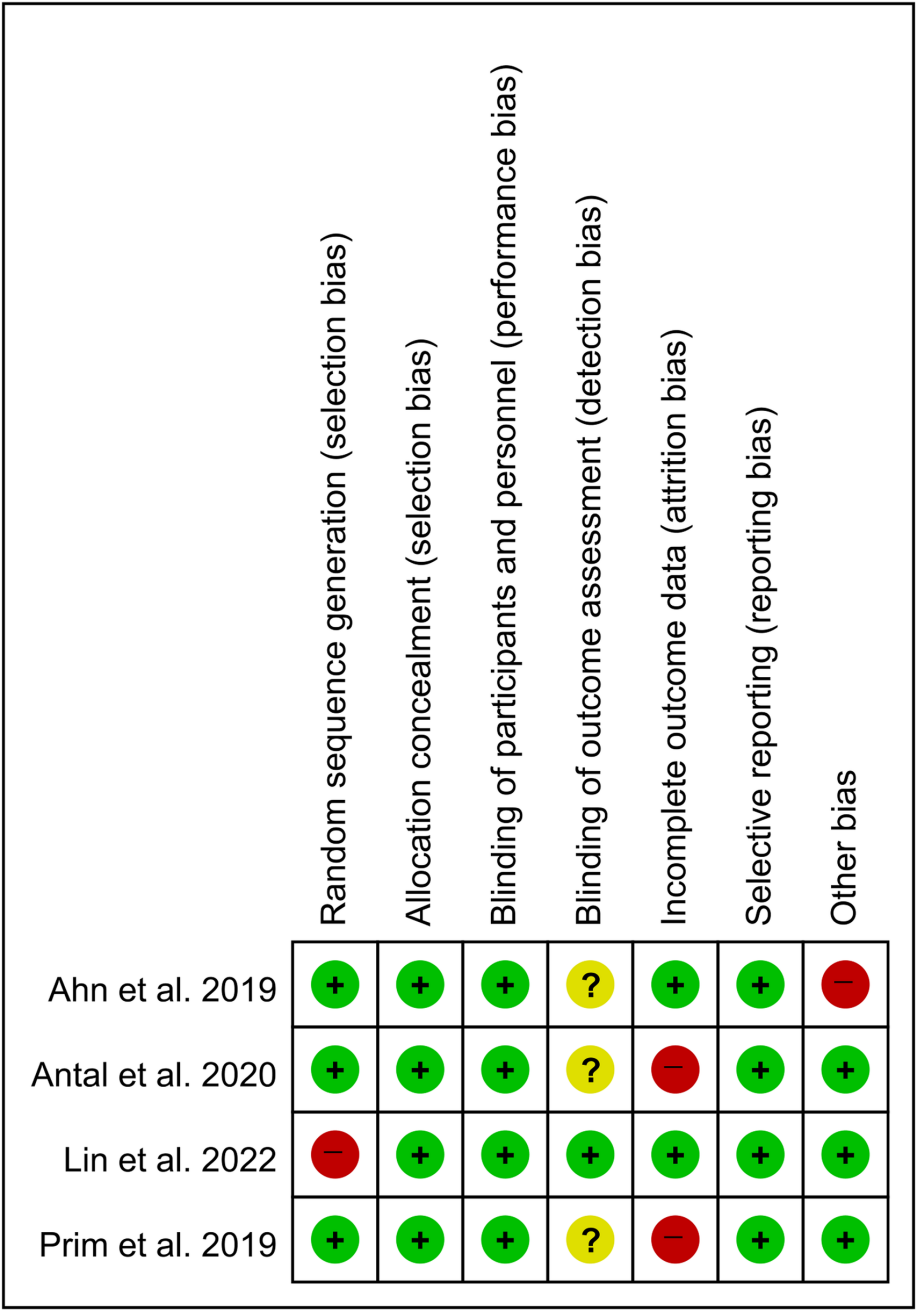
Risk of bias summary.

All four included studies had a low risk of bias in the domains of allocation concealment, blinding of participants and personnel, and selective reporting (Figure 2). In random sequence generation, three studies had a low risk of bias. In the domain of blinding outcome assessment, only one study had a low risk of bias, and in the domain of incomplete outcome data, two studies had a low risk of bias. Three studies had a low risk of bias in the domain of other biases. Among the 28 domains across all four included studies, 21 domains (75.0%) were determined to be low risk. The inter-rater agreement for the determination of potential bias of each study was 0.900 (*p* < 0.001) according to the kappa index.

## Discussion

In this review of tACS for chronic pain, we found four published articles in that met our inclusion criteria (13, 14, 16, 17). The patients included in the previous studies had fibromyalgia in one study (16), LBP in two studies (13, 17), and migraine in one study (14).

Patients with chronic pain exhibit impaired alpha oscillations (13, 19). It is known that pain perception suppresses alpha oscillations (13, 19). Previous studies found that the amplitude of alpha oscillations in the brain was negatively correlated with the severity of chronic pain (20–22). tACS can modulate neural oscillations by applying oscillating electrical currents (10–12). The enhanced alpha oscillations by tACS can be suggested to induce pain relief. Additionally, in patients with chronic pain, abnormal activation of the thalamic nuclei, insula, anterior cingulate, and sensory and prefrontal cortices was observed during pain processing (23, 24). The previous studies using EEG found increased theta rhythm mainly located in the anterior cingulate and frontal cortex, which are part of the thalamocortical circuit. The thalamocortical circuit plays a crucial role in processing and transmitting pain signals in the human brain (25, 26). Abnormal activation of the thalamocortical circuit is considered a key pathology inducing the development of chronic pain (25, 26). Therefore, chronic pain is interpreted as the result of thalamocortical dysrhythmia (25). Low-intensity alternating currents produced by tACS could modulate abnormal neural activation within the thalamocortical circuit, which is supposed to be helpful for alleviating chronic pain (15).

However, contrary to our expectations, the results of our study that integrated the findings of four previous studies were conflicting (13, 14, 16, 17). In the study on fibromyalgia (16), it did not show a better pain-reducing effect of tACS compared with sham stimulation (16). The two studies on LBP showed opposite results (13, 17). The study of Ahn et al. (13) showed a positive pain-reducing effect of tACS, but study of Prim et al. (17) did not find any significant difference between the pain-reducing effect of tACS and that of sham stimulation. Regarding migraine, the study reported that tACS was helpful for terminating migraine attacks and reducing pain severity during migraine attacks (14). The conflicting results hinder the conclusion of the effectiveness of tACS for controlling chronic pain.

Responses to tACS can vary among individuals. Factors such as the brain anatomy, exact placement of electrodes, and brain state during stimulation would influence the pain-reducing effect of tACS (27). Also, patients suffering from pain exhibit a wide range of pain patterns and characteristics. The individual variation in tACS effects might have contributed to the conflicting results in previous studies.

Additionally, even if the previous studies demonstrated a positive pain-reducing effect of tACS (13, 14), patients with LBP reported only immediate effects after tACS sessions (13). The pain relief effect of tACS in patients with migraines lasted only 24–48 h following tACS treatment (14). The long-term effect of tACS was not reported or evaluated in the previous studies. Therefore, studies investigating the effect of tACS on controlling chronic pain are required.

In the previous studies, the number of stimulation sessions varied in each study. The study of Lin et al. (16) applied tACS with 10 sessions, but the other studies used tACS with a single session (13, 14, 17). Also, the brain area to which tACS was applied and the intensity of current stimulation varied across the previous studies (13, 14, 16, 17). Further research should be conducted to establish the most effective tACS treatment protocol for controlling chronic pain.

In this review, we investigated the effectiveness of tACS in controlling chronic pain. We reviewed four studies (13, 14, 16, 17), among which two reported a positive effect on pain control (13, 14), while the remaining two studies reported negative results (16, 17). Furthermore, even if the positive pain-reducing effect of tACS was reported in two studies, it was only an immediate or short-term pain-relieving effect (13, 14). We think that there is still insufficient research to draw a conclusive judgment on the effects of tACS on controlling chronic pain. For a definitive conclusion, more studies across various chronic pain-related diseases are required. Also, studies for determining the optimal stimulation area, intensity, duration, and frequency for tACS treatment should be conducted in the future.

## Author contributions

MC: Writing – original draft, Writing – review & editing, Conceptualization, Formal analysis, Project administration, Visualization, Investigation, Methodology, Resources. M-MB: Methodology, Writing – review & editing. MB-R: Formal analysis, Methodology, Validation, Writing – review & editing. SY: Conceptualization, Formal analysis, Project administration, Visualization, Writing – original draft, Writing – review & editing.

## References

[1] Chang MC. The blind spot and challenges in pain management. J Yeungnam Med Sci. (2022) 39:179–80. doi: 10.12701/jyms.2022.00339, PMID: 35678089 PMC9273138

[2] Lee JH Chang MC. Some suggestions for pain physicians working in real-world clinical settings. J Yeungnam Med Sci. (2023) 40:S123–4. doi: 10.12701/jyms.2023.00255, PMID: 37218143 PMC10718597

[3] Dahlhamer J Lucas J Zelaya C Nahin R Mackey S DeBar L. Prevalence of chronic pain and high-impact chronic pain among adults—United States, 2016. MMWR Morb Mortal Wkly Rep. (2018) 67:1001–6. doi: 10.15585/mmwr.mm6736a2, PMID: 30212442 PMC6146950

[4] Garland EL. Pain processing in the human nervous system: a selective review of nociceptive and biobehavioral pathways. Prim Care. (2012) 39:561–71. doi: 10.1016/j.pop.2012.06.013, PMID: 22958566 PMC3438523

[5] Sarnthein J Jeanmonod D. High thalamocortical theta coherence in patients with neurogenic pain. NeuroImage. (2008) 39:1910–7. doi: 10.1016/j.neuroimage.2007.10.019, PMID: 18060808

[6] Sarnthein J Stern J Aufenberg C Rousson V Jeanmonod D. Increased EEG power and slowed dominant frequency in patients with neurogenic pain. Brain. (2006) 129:55–64. doi: 10.1093/brain/awh631, PMID: 16183660

[7] Stern J Jeanmonod D Sarnthein J. Persistent EEG overactivation in the cortical pain matrix of neurogenic pain patients. NeuroImage. (2006) 31:721–31. doi: 10.1016/j.neuroimage.2005.12.042, PMID: 16527493

[8] Walton KD Dubois M Llinás RR. Abnormal thalamocortical activity in patients with complex regional pain syndrome (CRPS) type I. Pain. (2010) 150:41–51. doi: 10.1016/j.pain.2010.02.023 20338687

[9] May ES Hohn VD Nickel MM Tiemann L Gil Ávila C Heitmann H. Modulating brain rhythms of pain using transcranial alternating current stimulation (tACS)—a sham-controlled study in healthy human participants. J Pain. (2021) 22:1256–72. doi: 10.1016/j.jpain.2021.03.150 33845173

[10] Antal A Paulus W. Transcranial alternating current stimulation (tACS). Front Hum Neurosci. (2013) 7:317. doi: 10.3389/fnhum.2013.00317 23825454 PMC3695369

[11] Elyamany O Leicht G Herrmann CS Mulert C. Transcranial alternating current stimulation (tACS): from basic mechanisms towards first applications in psychiatry. Eur Arch Psychiatry Clin Neurosci. (2021) 271:135–56. doi: 10.1007/s00406-020-01209-9, PMID: 33211157 PMC7867505

[12] Wischnewski M Alekseichuk I Opitz A. Neurocognitive, physiological, and biophysical effects of transcranial alternating current stimulation. Trends Cogn Sci. (2023) 27:189–205. doi: 10.1016/j.tics.2022.11.013, PMID: 36543610 PMC9852081

[13] Ahn S Prim JH Alexander ML McCulloch KL Fröhlich F. Identifying and engaging neuronal oscillations by transcranial alternating current stimulation in patients with chronic low Back pain: a randomized, crossover, double-blind, sham-controlled pilot study. J Pain. (2019) 20:277.e1–277.e11. doi: 10.1016/j.jpain.2018.09.004 PMC6382517 30268803

[14] Antal A Bischoff R Stephani C Czesnik D Klinker F Timäus C. Low intensity, transcranial, alternating current stimulation reduces migraine attack burden in a home application set-up: a double-blinded, randomized feasibility study. Brain Sci. (2020) 10:888. doi: 10.3390/brainsci10110888 33233400 PMC7700448

[15] Bernardi L Bertuccelli M Formaggio E Rubega M Bosco G Tenconi E. Beyond physiotherapy and pharmacological treatment for fibromyalgia syndrome: tailored tACS as a new therapeutic tool. Eur Arch Psychiatry Clin Neurosci. (2021) 271:199–210. doi: 10.1007/s00406-020-01214-y, PMID: 33237361 PMC7867558

[16] Lin AP Chiu CC Chen SC Huang YJ Lai CH Kang JH. Using high-definition transcranial alternating current stimulation to treat patients with fibromyalgia: a randomized double-blinded controlled study. Lifestyles. (2022) 12:1364. doi: 10.3390/life12091364 PMC9506250 36143400

[17] Prim JH Ahn S Davila MI Alexander ML McCulloch KL Fröhlich F. Targeting the autonomic nervous system balance in patients with chronic low Back pain using transcranial alternating current stimulation: a randomized, crossover, double-blind. J Pain Res. (2019) 12:3265–77. doi: 10.2147/JPR.S208030 31849514 PMC6912089

[18] Higgins JPT Green S (2011). Cochrane handbook for systematic reviews of interventions. Version 5.1.0. Available at: http://handbook-5-1.cochrane.org/

[19] Arendsen LJ Henshaw J Brown CA Sivan M Taylor JR Trujillo-Barreto NJ. Entraining alpha activity using visual stimulation in patients with chronic musculoskeletal pain: a feasibility study. Front Neurosci. (2020) 14:828. doi: 10.3389/fnins.2020.00828, PMID: 32973429 PMC7468433

[20] Babiloni C Brancucci A Del Percio C Capotosto P Arendt-Nielsen L Chen AC. Anticipatory electroencephalography alpha rhythm predicts subjective perception of pain intensity. J Pain. (2006) 7:709–17. doi: 10.1016/j.jpain.2006.03.005 17018331

[21] Ploner M Sorg C Gross J. Brain rhythms of pain. Trends Cogn Sci. (2017) 21:100–10. doi: 10.1016/j.tics.2016.12.001, PMID: 28025007 PMC5374269

[22] Tu Y Zhang Z Tan A Peng W Hung YS Moayedi M. Alpha and gamma oscillation amplitudes synergistically predict the perception of forthcoming nociceptive stimuli. Hum Brain Mapp. (2016) 37:501–14. doi: 10.1002/hbm.23048, PMID: 26523484 PMC4843944

[23] Burgmer M Gaubitz M Konrad C Wrenger M Hilgart S Heuft G. Decreased gray matter volumes in the cingulo-frontal cortex and the amygdala in patients with fibromyalgia. Psychosom Med. (2009) 71:566–73. doi: 10.1097/PSY.0b013e3181a32da0, PMID: 19414621

[24] Staud R Craggs JG Perlstein WM Robinson ME Price DD. Brain activity associated with slow temporal summation of C-fiber evoked pain in fibromyalgia patients and healthy controls. Eur J Pain. (2008) 12:1078–89. doi: 10.1016/j.ejpain.2008.02.002, PMID: 18367419 PMC2582560

[25] Gücer G Niedermeyer E Long DM. Thalamic EEG recordings in patients with chronic pain. J Neurol. (1978) 219:47–61. doi: 10.1007/BF00313368, PMID: 81284

[26] Llinás RR Ribary U Jeanmonod D Kronberg E Mitra PP. Thalamocortical dysrhythmia: a neurological and neuropsychiatric syndrome characterized by magnetoencephalography. Proc Natl Acad Sci U S A. (1999) 96:15222–7. doi: 10.1073/pnas.96.26.15222 10611366 PMC24801

[27] Miyaguchi S Otsuru N Kojima S Yokota H Saito K Inukai Y. Gamma tACS over M1 and cerebellar hemisphere improves motor performance in a phase-specific manner. Neurosci Lett. (2019) 694:64–8. doi: 10.1016/j.neulet.2018.11.015, PMID: 30445151

