# Supplementary material for: Effectiveness of transcranial alternating current stimulation for controlling chronic pain: a systematic review
Source: Front Neurol. 2023 Dec 20;14:1323520. doi: 10.3389/fneur.2023.1323520 (PMC10773732; doi:10.3389/fneur.2023.1323520)
Supplement: Supplementary file 1 [file Table_1.DOCX]

Table 1. Characteristics of the included studies.

| **#** | **First author** | **Published year** | **No. of patient** | **Age, yr (mean ± SD)** | **Disease** | **Pain duration of patients** | **Study type** | **Outcome parameters** | **Results** |
| --- | --- | --- | --- | --- | --- | --- | --- | --- | --- |
| 1 | Lin (16) | 2022 | 35 (tACS:sham=18:17) | tACS: 48.3 ± 13.6  Sham: 48.9 ± 12.3 | Fibromyalgia | ≥3 months | RCT | NRS, FIQ, BAI, BDI-II, PSQI | - All the measured outcomes were not significantly different between the tACS and sham stimulation groups. |
| 2 | Ahn (13) | 2019 | 20 | Age range: 18-65, no information on mean | Low back pain | ≥5 months | Randomized, double-blind, crossover design | DVPRS, ODI, EEG | - The DVPRS score was significantly lower after tACS than after sham stimulation, but the ODI was not.  - The increase in alpha oscillations was significantly higher after tACS compared to sham stimulation. |
| 3 | Prim (17) | 2019 | 20 | 43.0 ± 13.4 | Low back pain | ≥6 months | Randomized, double-blind, crossover design | DVPRS | - No significant difference was found between tACS and sham stimulation. |
| 4 | Antal (14) | 2020 | 25 (tACS:sham=16:9) | tACS: 31.1 ± 8.9  Sham: 28.1 ± 10.5 | Migraine | ≥6 months | RCT | NRS | - The reduction of NRS at 2 and 4 hours after stimulation was significantly higher in the tACS group than in the sham stimulation group. |

*Abbreviations.* tACS, transcranial alternating current stimulation; SD, standard deviation; RCT, randomized controlled trial; NRS, numeric rating scale; FIQ, fibromyalgia impact questionnaire; BAI, Beck Anxiety Inventory; PSQI, Pittsburgh Sleep Quality Index; DVPRS, Defense and Veterans Pain Rating Scale; ODI, Oswestry Disability Index; EEG, electroencephalogram
