# Supplementary material for: Effectiveness of transcranial alternating current stimulation for controlling chronic pain: a systematic review
Source: Front Neurol. 2023 Dec 20;14:1323520. doi: 10.3389/fneur.2023.1323520 (PMC10773732; doi:10.3389/fneur.2023.1323520)
Supplement: Supplementary file 2 [file Table_2.DOCX]

Table 2. Application methods for tACS treatment and combined treatments with tACS.

| **#** | **First author** | **Simulation site** | **Intensity (mA)** | **Duration**  **(min)** | **Frequency (Hz)** | **No. of sessions** | **Combined treatments with tACS** | **Treatment received by control group** |
| --- | --- | --- | --- | --- | --- | --- | --- | --- |
| 1 | Lin (16) | M1 | 1 | 20 | 50 | 10 | No combined treatment. | Sham stimulation |
| 2 | Ahn (13) | Bilateral F-lobe (F3, F4) | 1 | 40 | 10 | 1 per study arm | No combined treatment. | Sham stimulation |
| 3 | Prim (17) | M1 | 1 | 40 | 10 | 1 per study arm | No combined treatment. | Sham stimulation |
| 4 | Antal (14) | O-lobe (Oz) | 0.4 | 15 | 140 | 1 (at the beginning of the migraine attack) | The included patients were allowed to take their regular acute migraine medications | Sham stimulation & regular acute migraine medications |

*Abbreviations.* tACS, transcranial alternating current stimulation; M1, primary motor cortex; F-lobe, frontal lobe; O-lobe, occipital lobe
