# Supplementary material for: Effectiveness of transcranial alternating current stimulation for controlling chronic pain: a systematic review
Source: Front Neurol. 2023 Dec 20;14:1323520. doi: 10.3389/fneur.2023.1323520 (PMC10773732; doi:10.3389/fneur.2023.1323520)
Supplement: Supplementary file 3 [file Table_3.DOCX]

**Search terms and strategies**

**Search strategy for PubMed**

#1 Search: "Transcranial Alternating Current Stimulation" OR "tACS"

"Transcranial Alternating Current Stimulation"[All Fields] OR "tACS"[All Fields]

#2 Search: "pain"

"pain"[All Fields]

#3 Search: #1 AND #2 ("Transcranial Alternating Current Stimulation"[All Fields] OR "tACS"[All Fields]) AND "pain"[All Fields]

**Search strategy for Embase**

#1 'transcranial alternating current stimulation'/exp OR 'transcranial alternating current stimulation' OR 'tacs'

#2 'pain'/exp OR 'pain'

#3 #1 AND #2 ('transcranial alternating current stimulation'/exp OR 'transcranial alternating current stimulation' OR 'tacs') AND ('pain'/exp OR 'pain')

**Search strategy for Scopus**

#1 ALL ("Transcranial Alternating Current Stimulation" OR "tACS")

#2 ALL ("pain")

#3 #1 AND #2 (ALL("Transcranial Alternating Current Stimulation" OR "tACS")) AND (ALL("pain"))

**Search strategy for Cochrane library**

#1 ("Transcranial Alternating Current Stimulation" OR "tACS") in Trials

#2 ("pain") in Trials

#3 #1 AND #2 in Trials (Word variations have been searched)

| **Database** | **Keywords** |
| --- | --- |
| PubMed | ("Transcranial Alternating Current Stimulation"[All Fields] OR "tACS"[All Fields]) AND "pain"[All Fields] 147 |
| Embase | ('transcranial alternating current stimulation'/exp OR 'transcranial alternating current stimulation' OR 'tacs') AND ('pain'/exp OR 'pain') 427 |
| Cochrane library | ("Transcranial Alternating Current Stimulation" OR "tACS") AND "pain" in Trials (Word variations have been searched) 227 |
| Scopus | ALL ( ( "Transcranial Alternating Current Stimulation" OR "tACS" ) AND "pain" ) 1529 |
